# LINE-1 Methylation Patterns as a Predictor of Postmolar Gestational Trophoblastic Neoplasia

**DOI:** 10.1155/2015/421747

**Published:** 2015-09-13

**Authors:** Ruangsak Lertkhachonsuk, Krissada Paiwattananupant, Patou Tantbirojn, Prakasit Rattanatanyong, Apiwat Mutirangura

**Affiliations:** ^1^Division of Gynecologic Oncology, Department of Obstetrics and Gynecology, Faculty of Medicine, Chulalongkorn University, King Chulalongkorn Memorial Hospital, Bangkok 10330, Thailand; ^2^Division of Gynecologic Pathology, Department of Obstetrics and Gynecology, Faculty of Medicine, Chulalongkorn University, King Chulalongkorn Memorial Hospital, Bangkok 10330, Thailand; ^3^Center of Excellence in Molecular Genetic of Cancer and Human Disease, Department of Anatomy, Faculty of Medicine, Chulalongkorn University, King Chulalongkorn Memorial Hospital, Bangkok 10330, Thailand

## Abstract

*Objective*. To study the potential of long interspersed element-1 (LINE-1) methylation change in the prediction of postmolar gestational trophoblastic neoplasia (GTN). *Methods*. The LINE-1 methylation pattern from first trimester placenta, hydatidiform mole, and malignant trophoblast specimens were compared. Then, hydatidiform mole patients from 11999 to 2010 were classified into the following 2 groups: a remission group and a group that developed postmolar GTN. Specimens were prepared for a methylation study. The methylation levels and percentages of LINE-1 loci were evaluated for their sensitivity, specificity, and accuracy for the prediction of postmolar GTN. *Results*. First, 12 placentas, 38 moles, and 19 malignant trophoblast specimens were compared. The hydatidiform mole group had the highest LINE-1 methylation level (*p* = 0.003) and the ^u^C^u^C of LINE-1 increased in the malignant trophoblast group (*p* ≤ 0.001). One hundred forty-five hydatidiform mole patients were classified as 103 remission and 42 postmolar GTN patients. The %^m^C^u^C and %^u^C^m^C of LINE-1 showed the lowest *p* value for distinguishing between the two groups (*p* < 0.001). The combination of the pretreatment *β*-hCG level (≥100,000 mIU/mL) with the %^m^C^u^C and %^u^C^m^C, sensitivity, specificity, PPV, NPV, and accuracy modified the levels to 60.0%, 92.2%, 77.4%, 83.8%, and 82.3%, respectively. *Conclusions*. A reduction in the partial methylation of LINE-1 occurs early before the clinical appearance of malignant transformation. The %^m^C^u^C and %^u^C^m^C of LINE-1s may be promising markers for monitoring hydatidiform moles before progression to GTN.

## 1. Introduction

Hydatidiform mole, a genetic imprinting disease [1–3], is caused by fertilization abnormalities such as androgenetic (monospermic and dispermic) diploid or biparental triploid [4]. The incidence of this disease varies around the world. However, Southeast Asia still has a higher incidence than Western countries [5]. Most hydatidiform mole patients reach remission after primary treatment; however, 8–30% [6, 7] of patients develop postmolar gestational trophoblastic neoplasm (GTN). A high-risk hydatidiform mole is characterized by a human chorionic gonadotropin level (hCG) >100,000 mIU/mL, excessive uterine enlargement, and theca lutein cysts that are >6 cm in diameter. However, these clinical features are only able to predict 40% of postmolar GTN [8]. Currently, there is still no appropriate method for predicting malignant changes in hydatidiform moles.

Although hydatidiform moles can now be diagnosed earlier than in previous decades [9], the incidence of postmolar GTN is still unchanged from these earlier times. This suggests that the malignant potential of hydatidiform moles begin when the moles form. Thus, genetic factors may play a crucial role in the malignant transformation of hydatidiform moles. Investigation of molecular markers in hydatidiform moles may aid in the early prediction of postmolar GTN. Epigenetic change in cancer is an event that causes abnormal gene expression and promotes carcinogenesis, even when the DNA sequences do not change [10–14]. DNA methylation is one of the mechanisms in which methylated cytosines precede guanine areas, which are called CpG island. Two common methylation changes in cancer are promoter hypermethylation and genome-wide hypomethylation at interspersed repetitive sequences (IRS) or transposon-derived sequences. The role of promoter hypermethylation is to inhibit tumor suppressor gene functions. Loss of IRS methylation leads to several consequences including genomic instability and genome-wide gene expression changes [15–18].

The methylation status of long interspersed element-1 (LINE-1) in cancer has been reported in many cancers [15]. LINE-1 is an interspersed repetitive sequence in the human genome, and elements of methylation have been used to represent genome-wide methylation [18]. Recent evidence has demonstrated LINE-1 hypomethylation in several cancers including head and neck cancer, breast cancer, bladder cancer, hepatic cancer, lung cancer, prostate cancer, colon cancer, and gynecologic cancer [8, 19–23]. In most cancers, LINE-1 methylation levels are lower than in normal tissues. Interestingly, alterations in DNA methylation are not randomly distributed in partial hydatidiform moles (PHMs). Perrin et al. reported global hypomethylation, LINE-1 hypermethylation, and unchanged methylation in PHMs [24]. We aimed to explore the IRS methylation levels and patterns of GTN as well as investigate the role of LINE-1 methylation in the prediction of postmolar GTN in hydatidiform mole patients.

For this reason, we evaluated the methylation statuses of IRS using Combined Bisulfite Restriction Analysis (COBRA). Unlike other techniques, COBRA differentiates IRS sequences into the following 4 methylation-status categories: hypermethylated, hypomethylated, and 2 forms of partially methylated loci. COBRA also provides information on the methylation levels [25, 26]. These subclassifications improved the sensitivity of the test in early cancer detection over other techniques, revealing only the overall methylation levels such as pyrosequencing. Recently, we reported that the LINE-1 hypomethylated loci distinguish tumor DNA more efficiently than the overall methylation levels [27, 28]. Moreover, while there were no LINE-1 methylation level changes in the oral epithelium of smokers, LINE-1s of partially methylated loci were different [29]. Therefore, the alteration in the percentage of the LINE-1 partially methylated loci may indicate early genome-wide hypomethylation in the multistep process of carcinogenesis.

## 2. Materials and Methods

This study was approved by the institutional review board of the Faculty of Medicine, Chulalongkorn University, Bangkok, Thailand. Pathological specimens were retrieved between 1999 and 2010. Patients' demographic and clinical data were reviewed from medical records.

### 2.1. Collection of Specimens

First, we studied the differences in the LINE-1 methylation levels among first trimester placenta (*n* = 12), hydatidiform moles (*n* = 38), and malignant trophoblasts (invasive mole and choriocarcinoma) (*n* = 19). Formalin-fixed, paraffin-embedded (FFPE) specimens were randomly collected from the Gynecologic Pathology Unit, King Chulalongkorn Memorial Hospital. Then, patients with hydatidiform moles who had been treated between 1999 and 2010 were recruited, and we reviewed these patients' medical records. The demographic data of the patients, including age, obstetrics history, histology, serum hCG level, and treatment outcomes, were collected. Postmolar GTN was defined by the FIGO criteria [30]. FFPE specimens from these patients were processed to analyze the methylation levels and patterns to be used as diagnostic tool for the malignant transformation of hydatidiform moles.

One gynecologic pathologist reviewed the hematoxylin and eosin-stained slides for all the sections, verified the quality of tissue, and mapped the studied areas. The expression of p57, observed by immunohistochemistry, was determined to differentiate between complete and partial mole. Unavailable paraffin-embedded specimens and degenerated tissue were excluded from this study. Paraffin-embedded specimens were collected and prepared at a 5 *μ*m thickness on the slides. The slides were deparaffinized with xylene solution and absolute alcohol. Microdissection was then performed by the laser caper technique. Lysis buffer was added to mix the microdissected tissues in micropipette tubes. DNA was then separated from other proteins by using phenol-chloroform-isoamyl alcohol.

### 2.2. DNA Extraction and COBRA LINE-1 PCR

**Figure 1:**
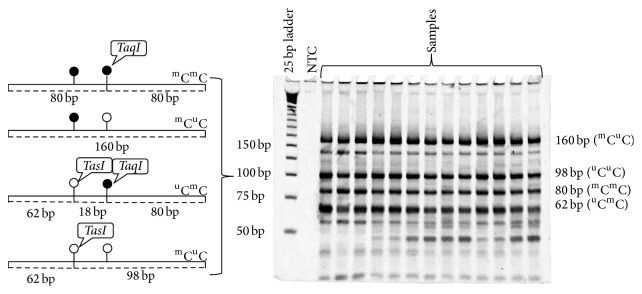
Methylation patterns of the LINE-1 methylation patterns of COBRA LINE-1. The following four patterns of methylated CpGs were demonstrated: hypermethylation (^m^C^m^C), hypomethylation (^u^C^u^C), and two forms of partial methylation (^m^C^u^C and ^u^C^m^C).

DNA extraction and PCR were performed by the COBRA LINE-1 protocol [20, 31]. Briefly, 22 M NaOH was used for the denaturing of genomic DNA at 37°C for 10 minutes. DNA was then treated with 20 *μ*L of 10 mM hydroquinone and 520 *μ*L of 3 M sodium bisulfite at 50°C for 16–20 hours to convert the unmethylated cytosine to uracil. DNA was purified and incubated in 0.33 M NaOH at 25°C for 3 min, ethanol precipitated, washed with 70% ethanol, and resuspended in 20 *μ*L of H_2_O. Two microliters of bisulfite DNA was annealed with two added primers for COBRA LINE-1, 5-CCGTAAGGGGTTAGGGAGTTTTT-3 and 5-RTAAAACCCTCCRAACCAAATATAAA-3, at 50°C. Amplification of PCR was conducted for 40 cycles. LINE-1 amplicons (160 bp) were digested in 10 *μ*L reaction volumes with 8 U of* TasI* in 1x* TaqI* buffer (MBI Fermentas, Burlington, ON, Canada) at 65°C overnight and were then electrophoresed in 8% nondenaturing polyacrylamide gel. There were 4 bands on the electrophoresis of LINE-1: 160 bp (^m^C^u^C), 98 bp (^u^C^u^C), 80 bp (^m^C), and 62 bp (^u^C) (Figure 1). The intensities of the DNA fragments were measured twice by PhosphorImager using Image-Quant software (Molecular Dynamics, Sunnyvale, CA).

Recently, Pobsook et al. [25] found that the 160 bp uncut band is one of the partially methylated bands. Therefore, this study improved the LINE-1 methylation formula for COBRA LINE-1. The percentage of LINE-1 hypomethylated loci (^u^C^u^C) was calculated by LINE-1 formulas. The intensity of each band was divided by the length (bp) of the double-stranded DNA before the calculations were performed (*A* = %160/160, *B* = %98/94, *C* = %80/78, and *D* = %62/62).

The LINE-1 formula was calculated as the %^m^C (total methylation) = 100 × (*C* + *A*)/(*C* + *A* + *A* + *B* + *D*), % number of  ^m^C^m^C (hypermethylated loci) = 100 × ((*C* − *D* + *B*)/2/(*C* − *D* + *B*/2) +  *D* + *A*), %PM (partial methylation) = 100 × (*A* + *D* − *B*)/((*C* − *D* + *B*)/2 +  *A* + *D*), %^m^C^u^C (partial methylated loci) = 100 × (*A*/((*C* − *D* + *B*)/2) +  *D* + *A*), %^u^C^m^C (partial methylated loci) = 100 × ((*D* − *B*)/((*C* − *D* + *B*)/2) +  *D* + *A*), and %^u^C^u^C (hypomethylated loci) = 100 × (*B*/((*C* − *D* + *B*)/2) +  *D* + *A*). The same preparations of DNA from* HeLa*,* Daudi*, and* Jurkat* cell lines were used as positive controls in every experiment to adjust for interassay variation.

### 2.3. Statistical Analysis

The mean difference in the percentage of LINE-1 among the normal first trimester placenta, hydatidiform mole, and cancer group (invasive mole and choriocarcinoma) was analyzed using a one-way ANOVA. In the latter portion of the study, an ROC curve was created according to each group's percentage of methylation (^m^C), percentage of partially methylated loci (^m^C^u^C, ^u^C^m^C), and percentage of the hypomethylated loci (^u^C^u^C) to estimate the respective cut-off points. The sensitivity, specificity, positive predictive value (PPV), negative predictive value (NPV), and accuracy were calculated. The mean differences in the percentage levels between the remission group and malignant transformation group were analyzed by independent samples *t*-test. Statistical analysis was performed by SPSS software for Windows version 17.0 (SPSS Inc., Chicago, IL), and statistical significance was set at *p* values of less than 0.05.

## 3. Results

### 3.1. LINE-1 Methylation in 3 Different Trophoblastic Tissues (First Trimester Placenta, Hydatidiform Moles, and Malignant Trophoblast)

**Figure 2:**
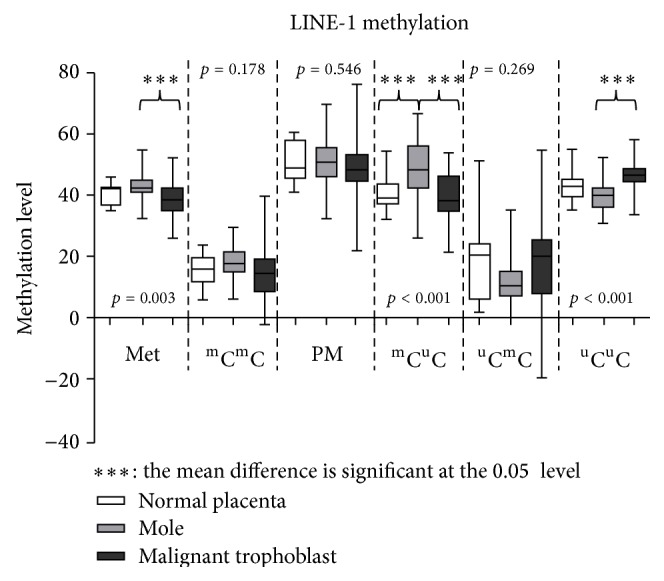
LINE-1 methylation in normal placenta, hydatidiform mole, and malignant trophoblast samples. Hydatidiform moles had the highest value in the mean total methylation (%^m^C) (*p* = 0.003) and hypermethylation (%^m^C^m^C) (*p* = 0.178). Malignant trophoblasts had significantly higher mean hypomethylation (%^u^C^u^C) (*p* < 0.001).

The differences in the LINE-1 methylation levels among the first trimester placenta group (*n* = 12), hydatidiform mole group (*n* = 38), and malignant trophoblast group (invasive mole and choriocarcinoma) (*n* = 19) are shown in Figure 2. The hydatidiform mole group had the highest value in the mean %^m^C (LINE-1 43.0% ± 3.8%, *p* = 0.003) and %^m^C^m^C (LINE-1 18.1% ± 5.1%, *p* = 0.178). The malignant trophoblast group had a significantly higher %^u^C^u^C than the hydatidiform mole group (LINE-1 47.2% ± 6.7% versus 40.0% ± 4.7%, *p* < 0.001).

### 3.2. LINE-1 Methylation in the Hydatidiform Mole, Comparing the Remission and Postmolar GTN Groups

In the study period, 145 hydatidiform mole patients were classified as 103 patients in the remission group and 42 patients in the postmolar GTN group. The ages in most cases were ≤40 years (128 cases, 88.30%). Pretreatment hCG levels over 100,000 mIU/mL were found in 82 cases (63.10%). Most (86.90%) cases were diagnosed with a complete hydatidiform mole (CHM). The incidence rates of malignant transformation were 33.33% and 5.27% for CHM and PHM cases, respectively. The mean age in the postmolar GTN group was older than the remission group (31.5 versus 27.8, *p* = 0.04). All postmolar GTN cases reached remission. Among these, 35 cases (87.50%) achieved successful treatment with single-agent chemotherapy. According to LINE-1 methylation, no significant difference was found with regard to patient age, pathological diagnosis, and metastasis. Only the pretreatment *β*hCG ≥ 100,000 mIU/mL group had a significantly higher %^u^C^u^C of LINE-1 than the pretreatment *β*hCG < 100,000 mIU/mL (47.2% ± 6.4% versus 44.1% ± 5.3%, *p* = 0.004) and a lower %PM (46.5% ± 8.9% versus 50.7% ± 7.7%, *p* = 0.005). The association of clinicopathologic variables and LINE-1 methylation level were demonstrated in Table 1.

**Figure 3:**
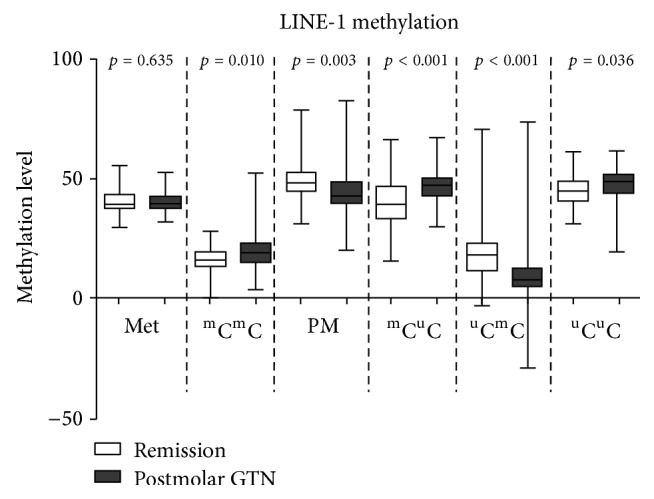
LINE-1 methylation patterns in the remission and postmolar GTN groups. The postmolar GTN group demonstrated a higher % hypermethylation (^m^C^m^C) (16.4% versus 19.8%, *p* = 0.010), % hypomethylation (^u^C^u^C) (45.2% versus 47.7%, *p* = 0.036), and %^u^C^m^C (20.7% versus 9.9%, *p* < 0.001). However, the remission group showed higher % partial methylation (49.4% versus 44.4%, *p* = 0.003) and %^m^C^u^C (40.7% versus 46.6%, *p* < 0.001).

When focused on the LINE-1 methylation levels between the remission hydatidiform mole and postmolar GTN, there were significant differences in these 2 groups with regard to the %PM (LINE-1 49.4% ± 7.5% versus 44.4% ± 9.5%, *p* = 0.003), %^m^C^m^C (LINE-1 16.4% ± 5.1% versus 19.8% ± 7.8%, *p* = 0.010), and %^u^C^u^C (LINE-1 45.2% ± 5.9% versus 47.7% ± 6.5%, *p* = 0.036). Furthermore, we found significant differences in the %^m^C^u^C (40.7% ± 10.4% versus 46.6% ± 6.6%, *p* < 0.001) and %^u^C^m^C (20.7% ± 13.6% versus 9.9% ± 13.5%, *p* < 0.001) in LINE-1 (Figure 3).

**Figure 4:**
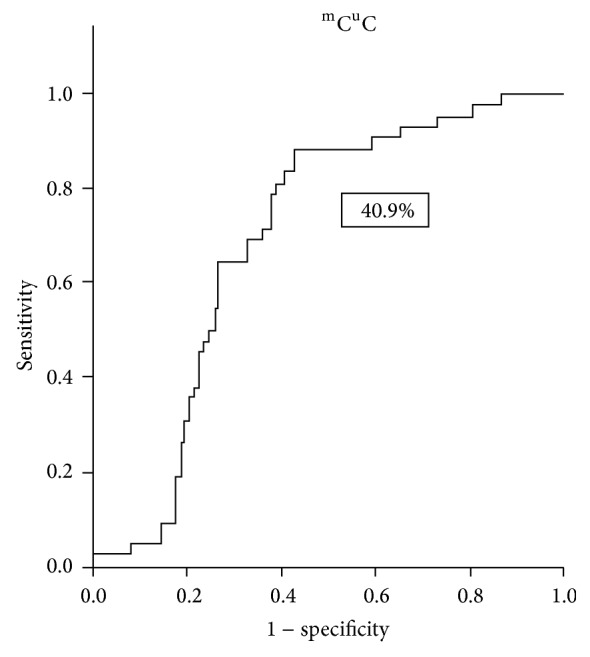
The ROC curve demonstrates %^m^C^u^C in LINE-1 if defined to positively test at ≥40.9%.

**Figure 5:**
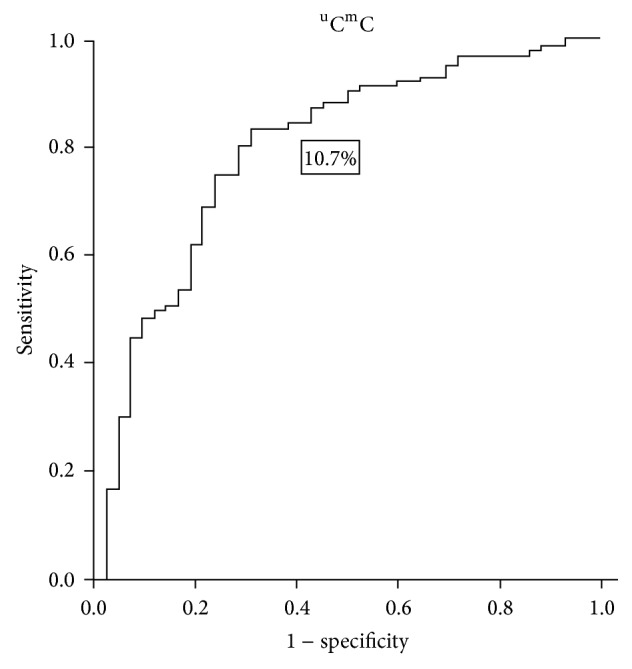
The ROC curve demonstrates the %^u^C^m^C in LINE-1 with cut-off level ≤10.7%.

The ROC curve of the %PM, %^m^C^u^C, and ^u^C^m^C in LINE-1 proved to be useful as a diagnostic tool. If the %^m^C^u^C in LINE-1 was defined to positively test at ≥40.9%, the sensitivity, specificity, PPV, NPV, and accuracy were 88.1%, 55.3%, 44.5%, 91.9%, and 64.8%, respectively (Figure 4). When the defined criterion of the %^u^C^m^C in LINE-1 was ≤10.7%, the sensitivity, specificity, PPV, NPV, and accuracy were 71.1%, 81.6%, 61.2%, 87.5%, and 78.6%, respectively (Figure 5). When the diagnosis was defined by both %^m^C^u^C and %^u^C^m^C as positive, the results were 69.0%, 85.4%, 65.9%, 87.1%, and 80.7% for the sensitivity, specificity, PPV, NPV, and accuracy, respectively. Furthermore, pretreatment with *β*hCG ≥ 100,000 mIU/mL had a significant difference in this study when the %^m^C^u^C and %^u^C^m^C were combined as a diagnostic tool plotted in an ROC curve. To be considered as a positive test, all pretreatment *β*hCG ≥ 100,000 mIU/mL and both %^m^C^u^C and %^u^C^m^C conditions had the same criteria. The sensitivity, specificity, PPV, NPV, and accuracy were then modified to 60.0%, 92.2%, 77.4%, 83.8%, and 82.3%, respectively (Table 2).

## 4. Discussion

Hydatidiform moles, particularly complete hydatidiform moles, have a risk of subsequent development of postmolar GTN. The mechanisms of this process are unknown. Many studies have demonstrated that an epigenetic mechanism may play a role in the malignant transformation of hydatidiform moles [6, 32]. Xue et al. [6] reported a study of 54 hydatidiform moles, 5 choriocarcinomas, and 10 first trimester placenta samples. Both hydatidiform mole and choriocarcinoma cases showed hypermethylation of the p16 gene, indicating that aberrant CpG island methylation is a frequent and likely disease-restricted occurrence in GTD. Li et al. [32] demonstrated hypermethylation of the SOX2 gene in 31/55 of hydatidiform mole cases and 4/4 of choriocarcinoma cases. Chen et al. [33] showed that both PHM and CHM have PTEN hypermethylation. Perrin et al. [24] also found LINE-1 hypermethylation in PHM. This study revealed results that LINE-1 hypomethylated loci (^u^C^u^C) levels were higher in choriocarcinoma and invasive moles, which was comparable with previous studies [8, 19–23].

In addition to genomic DNA mutation, amplifications, and deletions, DNA methylation also plays an important role in the process of carcinogenesis [34–36]. LINE-1 hypomethylation is a common epigenetic process in many cancer cells [21, 22, 37, 38]. The mechanisms of LINE-1 hypomethylation induce carcinogenesis, influence gene expression over the entire genome, and promote genomic instability. Hypomethylated intragenic LINE-1s are nuclear siRNA mediated cis-regulatory elements that can repress genes. This epigenetic regulation of retrotransposons likely influences many aspects of genomic biology [16]. In this study, we divided the partially methylated loci into two classes: ^m^C^u^C and ^u^C^m^C. The LINE-1 hypomethylation levels corresponded to significantly higher in cancer cells than in normal placenta and hydatidiform mole samples. The partially methylated loci numbers of ^m^C^u^C had significantly higher in hydatidiform moles than the normal placenta and malignant trophoblast samples. These findings suggest that methylation may play a role in multistep carcinogenesis. Interestingly, when we compared the LINE-1 expression between the remission hydatidiform mole group and postmolar GTN group, the percentage of LINE-1 overall partial methylation (PM) in the remission group was higher than the postmolar GTN group. However, there was a significantly higher ^m^C^u^C percentage of LINE-1 in the postmolar group. In contrast, the ^u^C^m^C percentage of LINE-1 was significantly higher in the remission group (Figure 2). Therefore, the loss of LINE-1 methylation in malignancy appears to be a multistep pattern.

Because prophylaxis chemotherapy showed a positive outcome for high-risk hydatidiform mole [39, 40], identifying patients with a higher risk of developing postmolar GTN is necessary. However, clinical indices were only 40–50% accurate [39, 40]. Therefore, more than half of these patients experienced toxicity from chemotherapy without any benefits. In the current study, we set up the ROC of the %^m^C^u^C and %^u^C^m^C to predict whether postmolar GTN would occur. Using a %^m^C^u^C level ≥40.9% and %^u^C^m^C level ≤10.7% combined with a pretreatment *β*hCG level (considering the pretreatment hCG level ≥100,000 mIU/mL as positive) has promising diagnostic power (sensitivity 60.0%, specificity 92.2%, PPV 77.4%, NPV 83.8%, and accuracy 82.3%). This diagnostic test may allow for the early detection of postmolar GTN and improve the quality of treatment.

In conclusion, a high level of %^m^C^u^C and a low level of %^u^C^m^C in LINE-1 were found in the postmolar GTN group. These findings occur early, before the clinical manifestations of malignant transformation, in hydatidiform moles. The precise measurement of the LINE-1 methylation level may be a promising marker in monitoring hydatidiform moles before progression to GTN.

## Figures and Tables

**Table 1:** Association of clinicopathologic variables and LINE-1 methylation level.

LINE-1 level (mean ± SD)	Parameters					
	^m^C	^m^C^m^C	PM	^m^C^u^C	^u^C^m^C	^u^C^u^C
Age (years)						
≤40	40.6 ± 4.0	17.2 ± 6.3	48.5 ± 8.1	42.1 ± 10.0	18.7 ± 14.7	45.5 ± 5.9
>40	39.7 ± 4.7	18.7 ± 5.4	44.2 ± 9.4	45.0 ± 7.8	9.1 ± 7.9	49.0 ± 7.9
*p* value	0.484	0.296	0.086	0.169	<0.001	0.094
Pretreatment *β*hCG level (mIU/mL)^*^						
<100,000	40.9 ± 4.1	16.1 ± 5.9	50.7 ± 7.7	43.3 ± 10.8	19.6 ± 14.8	44.1 ± 5.3
≥100,000	39.9 ± 3.7	17.7 ± 6.4	46.5 ± 8.9	42.1 ± 9.3	16.4 ± 14.7	47.2 ± 6.4
*p* value	0.173	0.133	0.005	0.544	0.234	0.004
Pathological diagnosis						
Complete hydatidiform mole	40.5 ± 4.0	17.5 ± 6.2	47.7 ± 8.1	43.0 ± 9.5	16.7 ± 13.7	46.0 ± 6.1
Partial hydatidiform mole	40.6 ± 4.8	16.5 ± 6.6	49.6 ± 10.3	38.7 ± 11.4	23.0 ± 17.8	45.4 ± 7.3
*p* value	0.939	0.565	0.463	0.137	0.158	0.748
Metastasis						
No metastasis	40.5 ± 4.2	20.0 ± 8.9	44.6 ± 10.7	47.0 ± 7.4	9.9 ± 16.1	47.3 ± 7.4
Metastasis	39.5 ± 2.5	18.9 ± 5.3	43.8 ± 7.2	46.2 ± 4.5	9.3 ± 6.0	48.8 ± 4.6
*p* value	0.304	0.578	0.806	0.660	0.860	0.415

PM = percentage of LINE-1 partial methylation.

^m^C^u^C, ^u^C^m^C = percentage of LINE-1 partially methylated loci.

^m^C^m^C = percentage of LINE-1 hypermethylated loci number.

^u^C^u^C = percentage of LINE-1 hypomethylated loci number.

^m^C = percentage of LINE-1 methylation.

^*^Incomplete data for 15 patients.

**Table 2:** Diagnostic power of the methylation levels combined with *β*hCG.

Diagnostic tools	PM	^m^C^u^C	^u^C^m^C	^m^C^u^C + ^u^C^m^C	^m^C^u^C + ^u^C^m^C + *β*hCG^*^
Sensitivity	60.9%	88.1%	71.1%	69.0%	60.0%
Specificity	84.5%	55.3%	81.6%	85.4%	92.2%
PPV	64.4%	44.5%	61.2%	65.9%	77.4%
NPV	87.0%	91.9%	87.5%	87.1%	83.8%
Accuracy	80.0%	64.8%	78.6%	80.7%	82.3%

*β*hCG-pretreatment *β*hCG: positive if ≥100,000 IU/mL.

PM-partial methylation: positive if ≤44.0%, ^m^C^u^C-partially methylated loci: positive if ≥40.9%, ^u^C^m^C-partially methylated loci: positive if ≤10.7%, and ^*^incomplete data in 15 patients.
